# Dual MAO A and HSP90 inhibitors enhance anti-PD-1 therapy and suppress colorectal cancer

**DOI:** 10.3389/fphar.2026.1866411

**Published:** 2026-08-24

**Authors:** Hui-Ju Tseng, Yueh-Lin Wu, Yan-Ling Chen, Chien-Hua Wang, Suddhasatwa Banerjee, Jing-Ping Liou

**Affiliations:** 1 School of Pharmacy, College of Pharmacy, Kaohsiung Medical University, Kaohsiung, Taiwan; 2 Division of Nephrology, Department of Internal Medicine, School of Medicine, College of Medicine, Taipei Medical University, Taipei, Taiwan; 3 TMU Research Center of Urology and Kidney, Taipei Medical University, Taipei, Taiwan; 4 Division of Nephrology, Department of Internal Medicine, Wan Fang Hospital, Taipei Medical University, Taipei, Taiwan; 5 School of Pharmacy, College of Pharmacy, Taipei Medical University, Taipei, Taiwan; 6 TMU Research Center for Drug Discovery, Taipei Medical University, Taipei, Taiwan

**Keywords:** CD8^+^ T cell infiltrations, colorectal cancer, dual MAO A/HSP90 inhibitors, immunotherapy resistance, MPT1B098, MPT1B099

## Abstract

**Introduction:**

Immune checkpoint inhibitors (ICIs) have revolutionized cancer therapy, yet their efficacy remains limited by tumor resistance, immune-related adverse events, and poor response in microsatellite stable colorectal cancer (CRC). To address these challenges, dual monoamine oxidase A (MAO-A) and heat shock protein 90 (HSP90) inhibitors, MPT1B098 and MPT1B099, were developed and evaluated for their therapeutic potential and immune-modulatory efficacy in CRC.

**Methods:**

Human and murine CRC cell lines were treated with MPT1B098 and MPT1B099 to assess their effects in cytotoxicity, cell migration, apoptosis, cell cycle arrest, and cell-surface PD-L1 expression. Western blotting was performed to evaluate key apoptotic and EMT-related markers. In vivo therapeutic efficacy and safety were evaluated using CT26 and MC38 subcutaneous syngeneic tumor models in BALB/c and C57BL/6 mice treated with MPT1B098, MPT1B099, and/or anti-PD1 antibodies. Tumor infiltration of CD8^+^ T cells and tissue histopathology were evaluated by immunohistochemistry and H&E staining.

**Results:**

In vitro, MPT1B098 and MPT1B099 exhibited potent cytotoxicity against human and murine CRC cell lines with sub-micromolar IC_50_ values, effectively inhibiting cell growth and migration while inducing apoptosis. Both compounds decreased cell-surface PD-L1 expression and modulated key apoptotic and EMT markers on Western blot. In vivo, both compounds were well tolerated at 10 mg/kg and significantly suppressed tumor growth in microsatellite instable and stable models. Notably, anti-PD1 monotherapy was effective only in MSI models, whereas MPT1B098 and MPT1B099 showed efficacy in both MSI and MSS tumors, with combination therapy producing a modest synergistic effect. Immunohistochemistry revealed increased CD8^+^ T cell infiltration following treatment, particularly with MPT1B099 in combination with anti-PD1 antibodies.

**Discussion:**

These findings highlight the dual anti-tumor and immune-modulatory properties of MPT1B098 and MPT1B099, mediated in part through PD-L1 downregulation and enhanced intratumoral T cell infiltration. Dual targeting of MAO-A and HSP90 represents a promising novel strategy to overcome immunotherapy resistance in MSS CRC and enhance the therapeutic efficacy of immune checkpoint blockade.

## 1 Introduction

The immune checkpoint proteins (ICP), expressed on the surface of immune cells, prevent immune cells from attacking normal cells by inhibiting immune cell activity and reducing the immune response. Tumors can exploit this “checkpoint” to evade immune system attack. This is an important strategy for tumor survival (Jiang et al., 2019). ICPs include: PD-1, CTLA-4, ICOS, LAG-3, TIGIT, BTLA, and TIM3 (Gaikwad et al., 2022). Among these, PD-1 (also known as CD279) was recognized as a key immune checkpoint that regulates T cells and B cells. Cancer cells escape from the immune system by utilizing the immune checkpoint (Jiang et al., 2019). Targeting the immune checkpoint is recognized as a novel and promising strategy for cancer treatment. This strategy has driven the development of 8 immune checkpoint inhibitors (ICIs) approved by the FDA for cancer treatment (Marin-Acevedo et al., 2021). The primary advantage of immunotherapy lies in its impact on the immune system, compared to traditional chemotherapy and targeted therapy. By blocking immunosuppressive signals and activating immune cells, ICIs restore the adaptive immune function and provide long-lasting effects (Makuku et al., 2021). However, only a limited number of patients benefit from immunotherapy.

ICIs have demonstrated great success, yet most patients show limited benefit, due to the tumor microenvironment with few immune cells, low antigen expression, and abundant (Sharma et al., 2017; Dobosz et al., 2022; Xie et al., 2020; Bonaventura et al., 2019). The limitations of ICIs also include immune-related adverse events, which are prevalent and sometimes fatal (Johnson et al., 2022). Another drawback of ICIs is the high cost of the immunotherapy, which can be a barrier for patients (Andrews, 2015). Microsatellite stability (MSS) and instability (MSI) critically influence immunotherapy outcomes in cancers such as gastric, pancreatic, and colorectal cancers (Li et al., 2020; Ratti et al., 2018; Ghidini et al., 2021). MSI (dMMR) tumors generate abundant neo-antigens that stimulate T cells and increase immune infiltration, but also upregulate checkpoint molecules like programmed cell death protein 1 ligand 1 (PD-L1). In contrast, MSS (pMMR) tumors have fewer mutations, limited immune infiltration, and low checkpoint expression, making ICIs less effective (Li et al., 2020; Ratti et al., 2018; Ghidini et al., 2021). However, MSI is less prevalent than MSS in most solid tumors (Hanse et al., 2016; Luchini et al., 2021). Take colorectal cancer (CRC) as an example, only 15% of patients have MSI CRC. The majority of CRC patients are MSS and show resistance to immunotherapy. Even in MSI patients, only about 40%–55% respond to ICIs (Sahin et al., 2019). These findings reveal the unmet medical need for MSS CRC. To overcome immunotherapy resistance, a combination of immune checkpoint inhibitors and other targeted agents is suggested (Liu et al., 2015; Hellmann et al., 2019; Eng et al., 2019; Callahan et al., 2017). These challenges suggest the need for an improved immune checkpoint inhibitor that overcomes current limitations.

The efficacy of ICIs is limited, as they can only benefit a small number of patients. Further, the tumor heterogeneity often contributes to drug resistance. Thence, we aim to develop a drug that can regulate the signal of the immune checkpoint, increase the cytotoxic molecules produced by tumor-infiltrating T cells, and also target other oncoproteins. We believe that a drug targeting multiple therapeutic targets might provide a more effective solution for regulating the tumor microenvironment and simultaneously reducing tumor growth.

**FIGURE 1:**
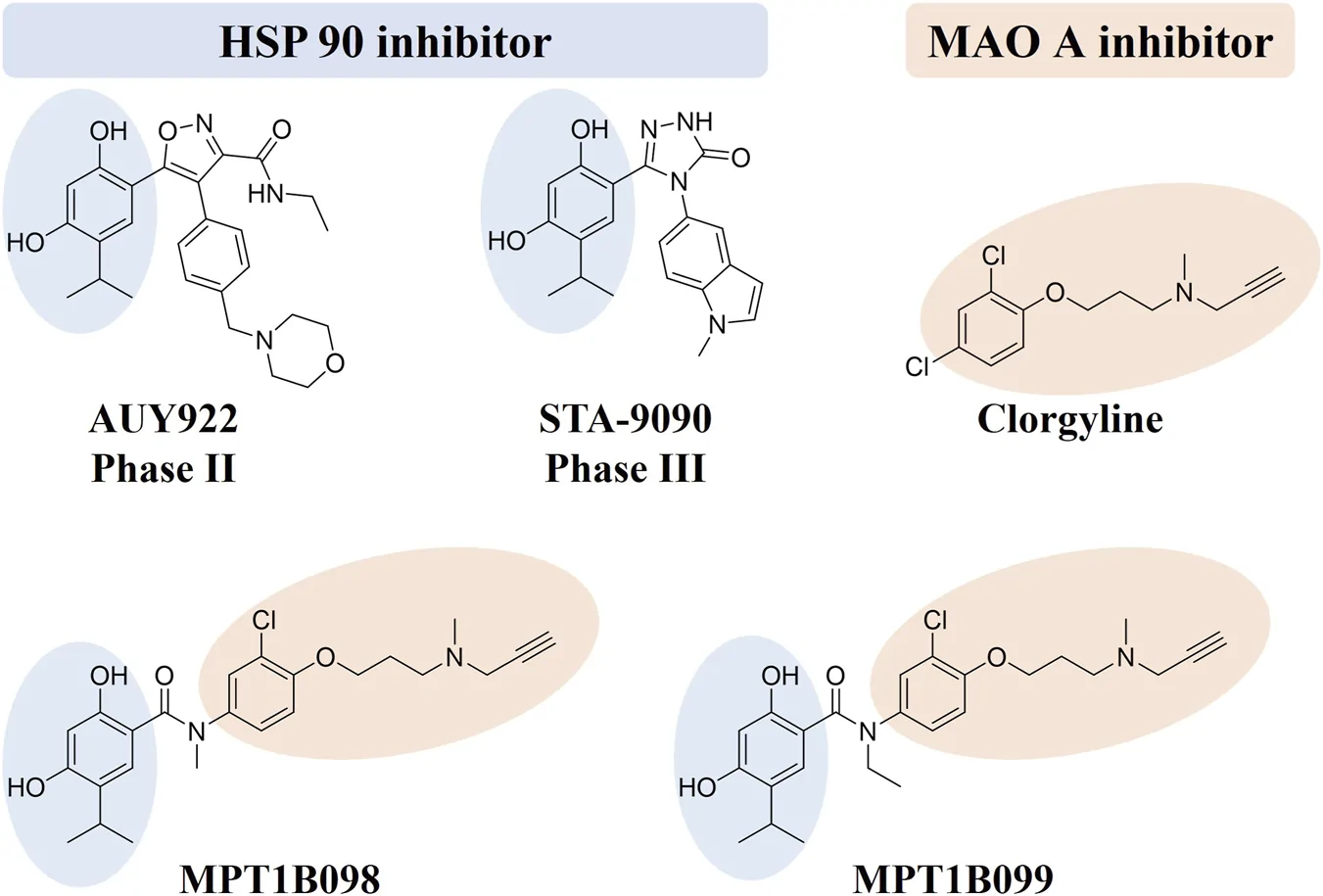
The structures of HSP90 inhibitors, MAO A inhibitor, and dual MAO S/HSP90 inhibitors–MPT1B098 and MPT1B099.

Previously, we developed dual MAO A and HSP90 inhibitors, MPT1B098 (4-b) and MPT1B099 (4-c), that inhibited glioblastoma growth *in vitro* and *in vivo* (Tseng et al., 2023). The structures of MPT1B098 and MPT1B099 were the combination of the reference MAO A inhibitor, clorgyline, and the HSP90 inhibitor, STA-9090 (Figure 1). The structures of MPT1B098 and MPT1B099 combined the 4-isopropylresorcinol pharmacophore of HSP90 inhibitors with the MAO A inhibitor, clorgyline scaffold, via amide linkers with methyl (MPT1B098) or ethyl (MPT1B099) substitutions, mimicking heterocyclic configurations of HSP90 inhibitors and employing a ring-opening concept to enhance their activity (Tseng et al., 2023). MPT1B098 (IC_50_ = 1.77 μM) showed better MAO A inhibition than MPT1B099 (IC_50_ = 4.58 μM), while MPT1B098 (IC_50_ = 16 nM) exhibited inferior effect in HSP90 inhibition compared to MPT1B099 (IC_50_ = 19 nM). Both compounds showed similar effects against glioblastoma cells (Tseng et al., 2023). HSP90 inhibition has been shown to decrease PD-L1 expression by regulating the master transcriptional regulators, as reported by Zavareh et al. (Zavareh et al., 2021). Hence, the effects of MPT1B098 and MPT1B099 in reducing PD-L1 expression have been evaluated in our previous research, and both compounds decreased PD-L1 expression in a dose-dependent manner (Tseng et al., 2023). The cell growth-inhibitory effects of MPT1B098 and MPT1B099 in NCI-60 cell lines have demonstrated their potential in colorectal cancer. Based on the aforementioned results, MPT1B098 and MPT1B099 have exhibited potency in immune regulation and CRC treatment (Tseng et al., 2023). However, the immune regulatory effects of MPT1B098 and MPT1B099 in CRC have not yet been assessed.

Based on our previous research, here we aimed to investigate the immune regulatory effects of MPT1B098 and MPT1B099 as potential next-generation immune checkpoint inhibitors. In this work, MPT1B098 and MPT1B099 were tested for cell growth inhibition and evaluated for their cytotoxicity, anti-migration, decreasing PD-L1 expression, and cell cycle arrest effects in colorectal cancer cells. The *in vivo* effects of MPT1B098 and MPT1B099 were also analyzed with colorectal cancer murine models, and their roles in regulating tumor-infiltrated CD8^+^ T cells were also assessed. Compared with PD-L1 antibodies, these compounds can reduce tumor growth and simultaneously reduce PD-L1 expression. These findings led to the identification of novel PD-L1 inhibitors with great potential in clinical drug development.

## 2 Materials and methods

### 2.1 Reagents

Sodium bicarbonate, sodium chloride, Tris, PEG400, and Triton X-100 were purchased from Sigma-Aldrich (Merck). STA-9090, Clorgyline, MPT1B098, and MPT1B099 were synthesized according to previously published procedures (purity >99%) (Tseng et al., 2023).

### 2.2 Cell culture

CT26 cells (Cat# 60447) were obtained from the Bioresource Collection and Research Center (BCRC, Hsinchu, Taiwan). MC38 cells (Cat# YC-A002) were obtained from UBIGENE (China). HT29 and HCT116 cells were kindly provided by Professor Ming-Jen Hsu, Taipei Medical University, which were purchased from American Type Culture Collection (ATCC, Manassas, VA, United States). Cells were maintained in DMEM (Thermo Fisher Scientific, Waltham, MA, United States; for HT29 and MC38), RPMI 1640 (Thermo Fisher Scientific; for CT26), and McCoy’s 5A (Sigma-Aldrich, Darmstadt, Germany; for HCT116) supplement with 10% fetal bovine serum (Thermo Fisher Scientific) and 1% antibiotic-antimycotic (penicillin, streptomycin, and amphotericin B, Thermo Fisher Scientific). All cells were maintained at 37 °C, 5% CO_2_ in a humidified atmosphere.

### 2.3 MTT assay

Cells were seeded in 3 × 10^3^ cells per well in 96-well plates. After the cells attached and reached a density of 30%–40% in the wells, they were treated with a compound-containing medium at various concentrations for 48 h. After 48 h, the medium was removed and replaced with medium containing 0.5 mg/mL MTT, and the cells were incubated for an additional 4 h. Following incubation, the MTT-containing medium was removed, and the formed MTT formazan was dissolved in 100 μL DMSO. The absorbance was measured at 595 nm. The data were plotted, and the IC_50_ values were calculated based on a sigmoidal dose-response equation using Graphpad Prism software.

### 2.4 Scratch assay

Cells were seeded in 24-well plates at a density of $1\times 10^{5}$ cell per well. After the cells attached and reached 80% confluence, a straight scratch was made in each well using a 1 mm pipette tip. Images of day 0 were taken using a microscope (SDPTOP XDF) and a 12.5 MP microscope camera (Mshot MSX2-C). After imaging, cells were incubated with the compound-containing medium for 24 h. After 24 h, images were taken again. The scratch distance was measured, and the percentage of closure was calculated.

### 2.5 Cell cycle arrest assay and cell apoptosis assay

Cells were seeded in 6-well plates at the density of $5\times 10^{4}$ cell per well. After cell attachment, cells were incubated with the compound-containing medium for 24 h. After incubation, cells were trypsinized and collected into tubes. For the cell cycle arrest assay, cells were fixed with 70% ice-cold ethanol for 30 min, washed twice with ice-cold PBS, treated with RNase A (Thermo Fisher Scientific), and stained with PI (Sigma-Aldrich). For the apoptosis assay, cells were stained with the eBioscience™ Annexin V Apoptosis Detection Kit (Thermo Fisher Scientific). The signals were analyzed using flow cytometry (BD FSCLyric). The data were analyzed with FlowJo.

### 2.6 Analysis of PD-L1 expression on cell surface

Cells were seeded in 6-well plates at the density of $5\times 10^{4}$ cell per well. After cell attachment, cells were incubated with the compound-containing medium for 24 h. After incubation, cells were trypsinized and collected into tubes. Cells were stained with a mouse anti-PD-L1 primary antibody (#14-5983-82, Thermo Fisher Scientific) for 1 h. After 1 h, cells were washed with PBS and incubated with rat anti-mouse antibody (#11-4015-82, Thermo Fisher Scientific). The signals were analyzed using flow cytometry (BD FSCLyric). The data were analyzed with FlowJo.

### 2.7 Western blot

Cells were seeded in 6-well plates at a density of $5\times 10^{5}$ cells per well. After cell attachment, cells were treated with a compound-containing medium for 24 or 48 h. After incubation, cells were washed with PBS, lysed with lysis buffer (20 mM Tris, 100 mM MgCl_2_, 125 mM NaCl, 1% Triton X-100) containing protease inhibitor cocktail (cOmpleteTM, Roche), and scraped with cell scrapers. The samples were centrifuged at 12,500 rpm to collect the protein and discard the cell debris. The proteins were separated with 8%–15% SDS-PAGE and transferred to PVDF membranes (Immun-Blot, Bio-Rad). The membranes were blocked with 5% nonfat milk in TBST and incubated with primary antibodies at room temperature for 2 h, including anti-GAPDH mAb (#60004-2-Ig, Proteintech), anti-E-cadherin pAb (#20874-1-AP, Proteintech), anti-Vimentin pAb (#10366-1-AP, Proteintech), anti-phospho-SMAD2 (Ser465, Ser467) (#44-244G, Invitrogen), anti-PARP1 (cleaved Asp214) mAb (#MA5-32104, Invitrogen), anti-PARP1 mAb (#MA5-15031, Invitrogen), anti-Caspase 3 mAb (#MA1-16843, Invitrogen), anti-cleaved caspase 3/P17/P19 (#82703-13-RR, Proteintech), anti-HSP70 mAb (#MA5-31961, Invitrogen), anti-phospho-PERK (Ser719) (#29546-1-AP, Proteintech), anti-phospho-AKT (Ser473) (#9271, Cell Signaling), and anti-phospho-EIF4E (Ser209) (#84981-1-RR, Proteintech). After washing the unbound antibodies, membranes were incubated with secondary antibodies for 1 h, goat anti-mouse IgG conjugated with HRP (#A28177, Invitrogen) and goat anti-rabbit IgG conjugated with HRP (#A27036, Invitrogen). After washing, the membranes were developed with enhanced chemiluminescent substrate (ECL, SuperSignal^TW^, West Pico PLUS, Thermo Fisher Scientific) and imaged with ChemiDoc™ MP Imaging System.

### 2.8 Tumor allograft model studies

Female BALB/c or C57BL/6 mice (10-weeks old, National Center for Biomodel (NCB), Taiwan) were subcutaneously injected with CT26 or MC38 ( $1\times 10^{6}$ cells per mouse) into the flanks of mice. When tumor size reached 50 mm^3^, mice were randomized into different groups of treatment, IgG control (BE0089, #919324F1, BioXcell), anti-PD1 antibodies (BE0146, #920823A2B, BioXcell), MPT1B098, MPT1B099, combined anti-PD1 and MPT1B098, and combined anti-PD1 and MPT1B099. IgG control and anti-PD1 were dissolved with normal saline in a concentration of 100 μg/100 μL per mouse, twice per week (Gouez et al., 2024). MPT1B098 and MPT1B099 were prepared with 5% DMSO, 67% PEG400, 28% normal saline in a concentration of 10 mg/kg, 80-100 uL per mouse, thrice per week. Body weight and tumor sized were measured thrice per week. Mice were euthanized after 14 days treatment, when tumors in the IgG control group reached 1500 mm^3^. Tumors were dissected and subjected for further studies.

### 2.9 Histological and immunohistochemistry analysis

The liver and tumor of Female BALB/C or C57BL/6 mice were dissected and fixed with 10% formalin after 14 days of treatment. The fixed tissues were subjected to NCB, further processed and embedded in paraffin. The tissue slides were sectioned at a thickness of 3–5 μm. The tissue slides were stained with hematoxylin and eosin (H&E), Masson’s trichrome (TRI), or anti-CD8 alpha pAb (#PA5-81344, Invitrogen), followed by secondary antibodies: goat anti-rabbit IgG conjugated with HRP (#31460, Invitrogen) using a standard procedure. The severity of lesions was analyzed by NCB and graded according to the method described by Shackelford et al. Degrees of lesions were graded histopathologically from one to five depending on severity (0 = not present, 1 = minimal <1%, 2 = slightly 1%–25%, 3 = moderate 26%–50%, 4 = moderately severe 51%–75%, 5 = severe/high 76%–100%). The signal of CD8 was quantified with ImageJ.

### 2.10 Data and statistical analysis

All *in vitro* experiments were conducted three times. Quantitative results were presented as the mean ± standard deviation (SD). Differences between the experimental and control groups were analyzed using one-way ANOVA tests. P < 0.05 was considered statistically significant compared to the respective control group.

## 3 Result

### 3.1 MPT1B098 and MPT1B099 reduce cell viability in MSI and MSS colorectal cancer cells

**FIGURE 2:**
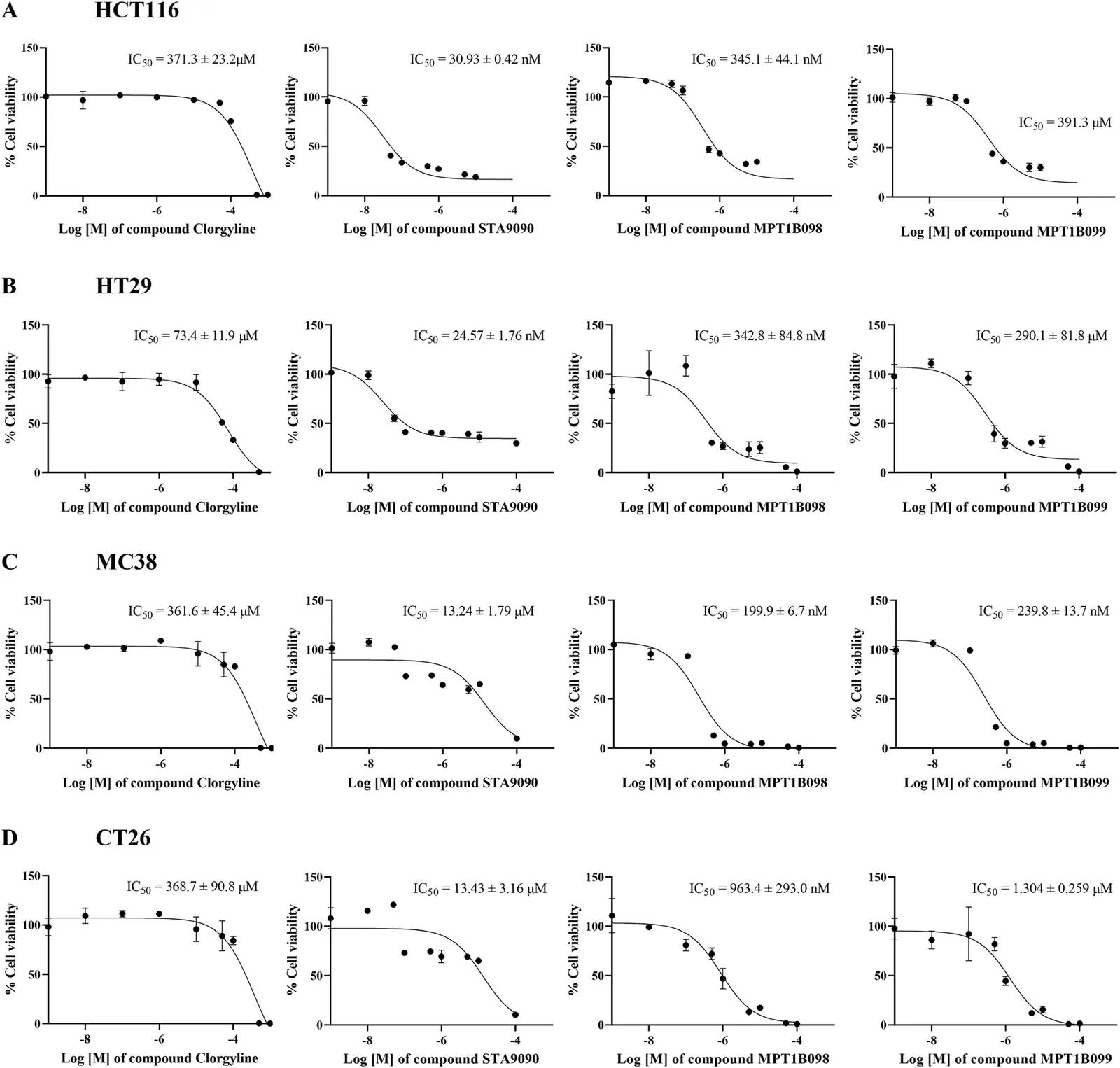
Cytotoxic effect of dual MAO A and HSP90 inhibitors against colorectal cancer cell lines. **(A)** HCT116, **(B)** HT29, **(C)** MC38, and **(D)** CT26 cells were treated with TGFβR1 inhibitors at the indicated dose for 48 h. After incubation, cell viability was assessed using the MTT assay. Each experiment was conducted in triplicate.

To assess the effects of MPT1B098 and MPT1B099 on the cell viability of CRC cells, both compounds were tested in two human CRC cell lines, HCT116 and HT29, and two murine CRC cell lines, CT26 and MC38. These human CRC cell lines exhibit distinct characteristics and correspond to two murine CRC cell lines. HCT116 and MC38 share similar profiles, including wild-type p53, reduced expression of the mismatch repair (MMR) gene, and exhibit microsatellite instability (MSI) (Gayet et al., 2001; Thomas et al., 2023). Conversely, HT29 and CT26 both display higher MMR expression and exhibit microsatellite stability (MSS) (Gayet et al., 2001; Thomas et al., 2023; Wu et al., 2024). Cell viability under treatment with MPT1B098, MPT1B099, and the reference compounds, clorgyline (a MAO A inhibitor), and STA-9090 (a HSP90 inhibitor), was evaluated using the MTT assay (Figure 2). Clorgyline demonstrated limited growth inhibition, with IC_50_ values ranging from 73 to 371 μM across all CRC cell lines. STA-9090 showed strong inhibitory effects in human CRC cells (IC_50_: 24–30 nM), but was markedly less potent in murine CRC cells (IC_50_: about 13 μM). Both MPT1B098 and MPT1B099 exhibited significant cytotoxicity against human and murine CRC cell lines. MPT1B098 showed comparable potency in HCT116 and HT29, with IC_50_ values 345.1 nM and 342.8 nM, respectively. MPT1B099 demonstrated slightly greater efficacy in HT29 (IC_50_: 290.1 nM) than in HCT116 (IC_50_: 391.3 nM). In murine CRC cells, both compounds were more effective against MC38 than CT26, with IC_50_ values of 199.9 and 239.8 nM for MC38, compared to 963.4 nM and 1.304 μM for CT26. These findings support further biological and mechanistic studies of MPT1B098 and MPT1B099 as promising candidates for CRC treatment.

### 3.2 MPT1B098 and MPT1B099 induce apoptosis in MSI and MSS colorectal cancer cells

**FIGURE 3:**
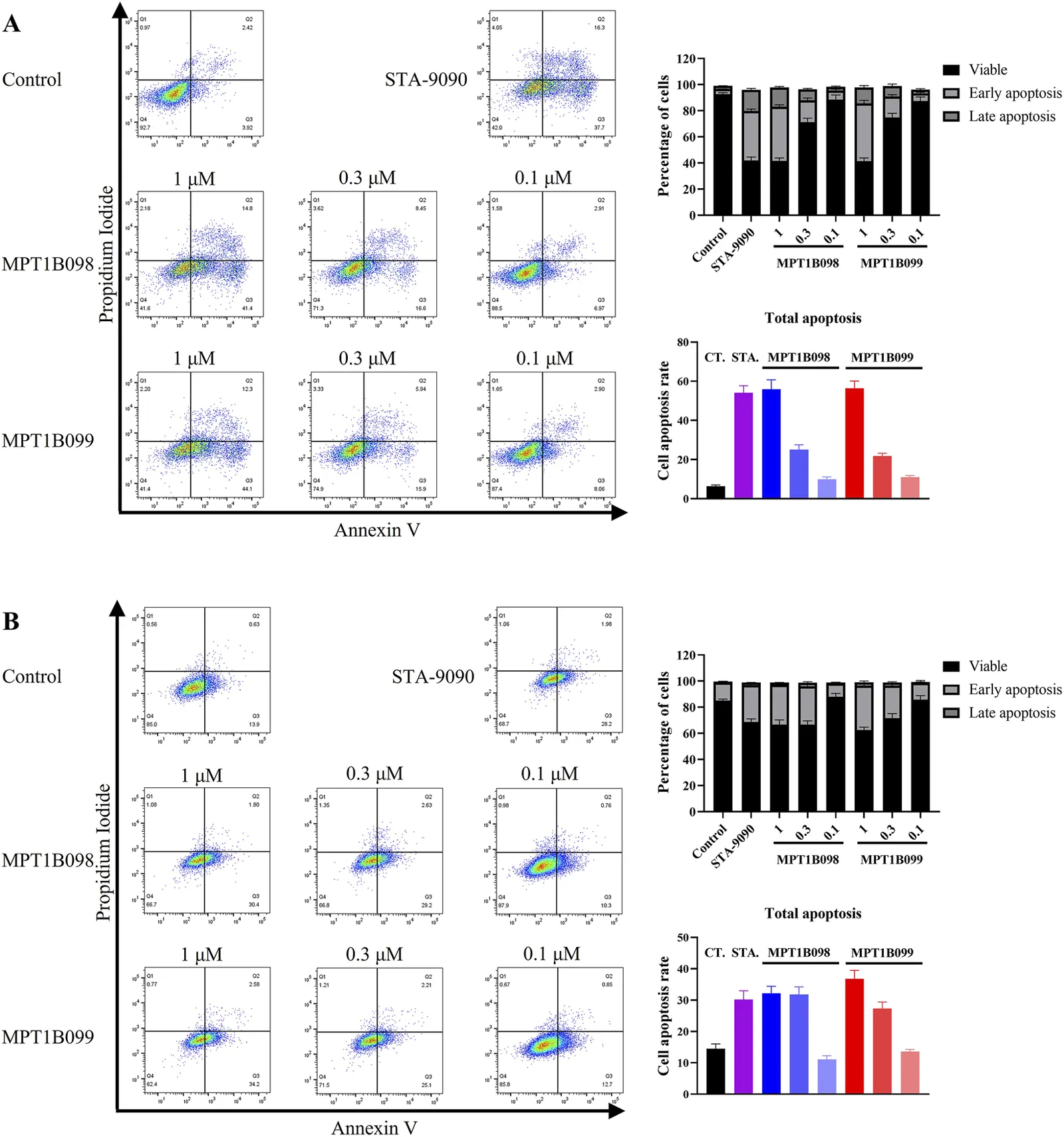
Apoptosis of colorectal cancer cells induced by MPT1B098 and MPT1B099. **(A)** HCT116 and **(B)** HT29 cells were treated with MPT1B098 and MPT1B099 at the indicated doses for 24 h. After incubation, the cells were trypsinized, stained with Annexin V and PI, and analyzed using flow cytometry. The Cell apoptosis rate was analyzed and quantified in the right bar graph. *p < 0.05, **p < 0.01, ***p < 0.001, ****p < 0.0001 vs. control.

Cancer cells typically express HSP90 at markedly elevated levels, approximately 2–10 times higher than in normal cells. The cancer-related client proteins of HSP90 play critical roles in promoting cancer cell growth, invasion, and resistance to treatment (Okamoto et al., 2008; Peng et al., 2022). Inhibition of HSP90 has been demonstrated to trigger cell cycle arrest and apoptotic cell death (Okamoto et al., 2008; Peng et al., 2022). Herein, the effects of MPT1B098 and MPT1B099 on apoptosis were assessed via Annexin V and PI staining (Figure 3). The percentage of cell population in early apoptosis was around 41% after treatment with 1 μM MPT1B098 or MPT1B099, 16% with 0.3 μM, and 6% with 0.1 μM, respectively. The percentage of cell population in late apoptosis was around 13% after treatment with 1 μM MPT1B098 or MPT1B099, 8% with 0.3 μM, and 3% with 0.1 μM, respectively. These results demonstrated that MPT1B098 and MPT1B099 could induce cell apoptosis in a dose-dependent manner, comparable to STA-9090.

### 3.3 MPT1B098 and MPT1B099 induce G2/M cell cycle arrest in MSI and MSS colorectal cancer cells

**FIGURE 4:**
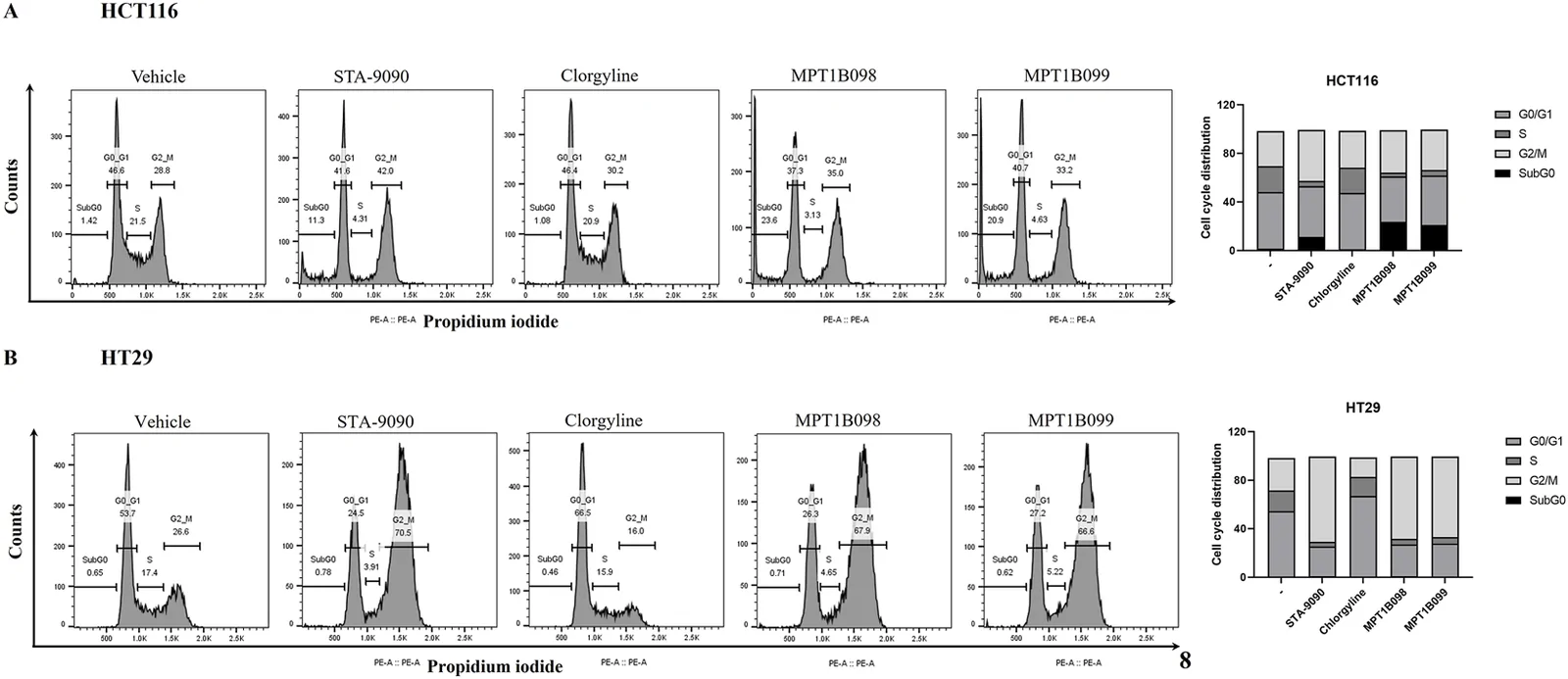
Cell cycle arrest of colorectal cancer cells induced by MPT1B098 and MPT1B099. **(A)** HCT116 and **(B)** HT29 cells were treated with MPT1B098, MPT1B099, and reference compounds at 1 μM for 24 h. After incubation, the cells were fixed, stained with PI, and analyzed using flow cytometry. The Cell apoptosis rate was analyzed and quantified in the right bar graph.

Inhibition of HSP90 has been shown to induce cell cycle arrest and apoptotic cell death (Okamoto et al., 2008; Peng et al., 2022; Senju et al., 2006). Similarly, inhibition of MAO A has been reported to affect cell growth, resulting in cell cycle arrest (Sblano et al., 2025). Accordingly, the cell cycle distribution of colorectal cancer cells following treatment with MPT1B098 and MPT1B099 was analyzed and quantified (Figure 4). In untreated HCT116 cells, the percentage of cells in the SubG0, G0/G1, S, and G2/M of normal cells was maintained at around 1.42%, 46.6%, 21.5%, and 28.8%, respectively; in HT29 cells, these values were 0.65%, 53.7%, 17.4%, and 26.6%. Upon treatment with MPT1B098, MPT1B099, and reference compound STA9090, the SubG0 cell population in HCT116 cells increased to 11.3%, 23.6%, and 20.9%, reflecting DNA fragmentation and apoptotic progression rather than checkpoint-mediated arrest. The G0/G1 population remained comparable to that of untreated cells, while the S phase population significantly decreased to 4.3%, 3.1%, and 4.6%. Only minor changes were observed in the G2/M phase population of HCT116 cells. In contrast, HT29 showed no increase in the SubG0 cell population following treatment. However, the S phase population markedly decreased to 3.9%, 4.7%, and 5.2% after treatment with STA-9090, MPT1B098, and MPT1B099, respectively. Notably, the G2/M phase population increased substantially to 70.5%, 67.9%, and 66.6%, compared to 26.6% in untreated cells.

HCT116, an MSI-high/dMMR cell line, with defective mismatch repair and impaired checkpoint signaling, is prone to bypass G2/M arrest and undergo apoptosis, leading to SubG0 accumulation. In contrast, HT29, an MSS/pMMR cell line with intact mismatch repair and checkpoint control, undergoes pronounced G2/M arrest upon treatment (Jain et al., 2015). Consistently, treatment with MPT1B098, MPT1B099, and reference compound STA9090 increased the SubG0 population in HCT116, reflecting apoptosis, whereas HT29 exhibited marked G2/M accumulation, indicative of mitotic arrest. These results demonstrate that mismatch repair status critically affects cell cycle fate after compound treatment, with dMMR cells favoring apoptosis and pMMR cells undergoing checkpoint-mediated arrest. Together, these findings indicated that MPT1B098 and MPT1B099 modulated cell cycle progression in colorectal cancer cells, inducing G2/M arrest in pMMR cells and promoting DNA fragmentation consistent with apoptosis in dMMR cells.

### 3.4 MPT1B098 and MPT1B099 decrease the migration of MSI and MSS colorectal cancer cells

**FIGURE 5:**
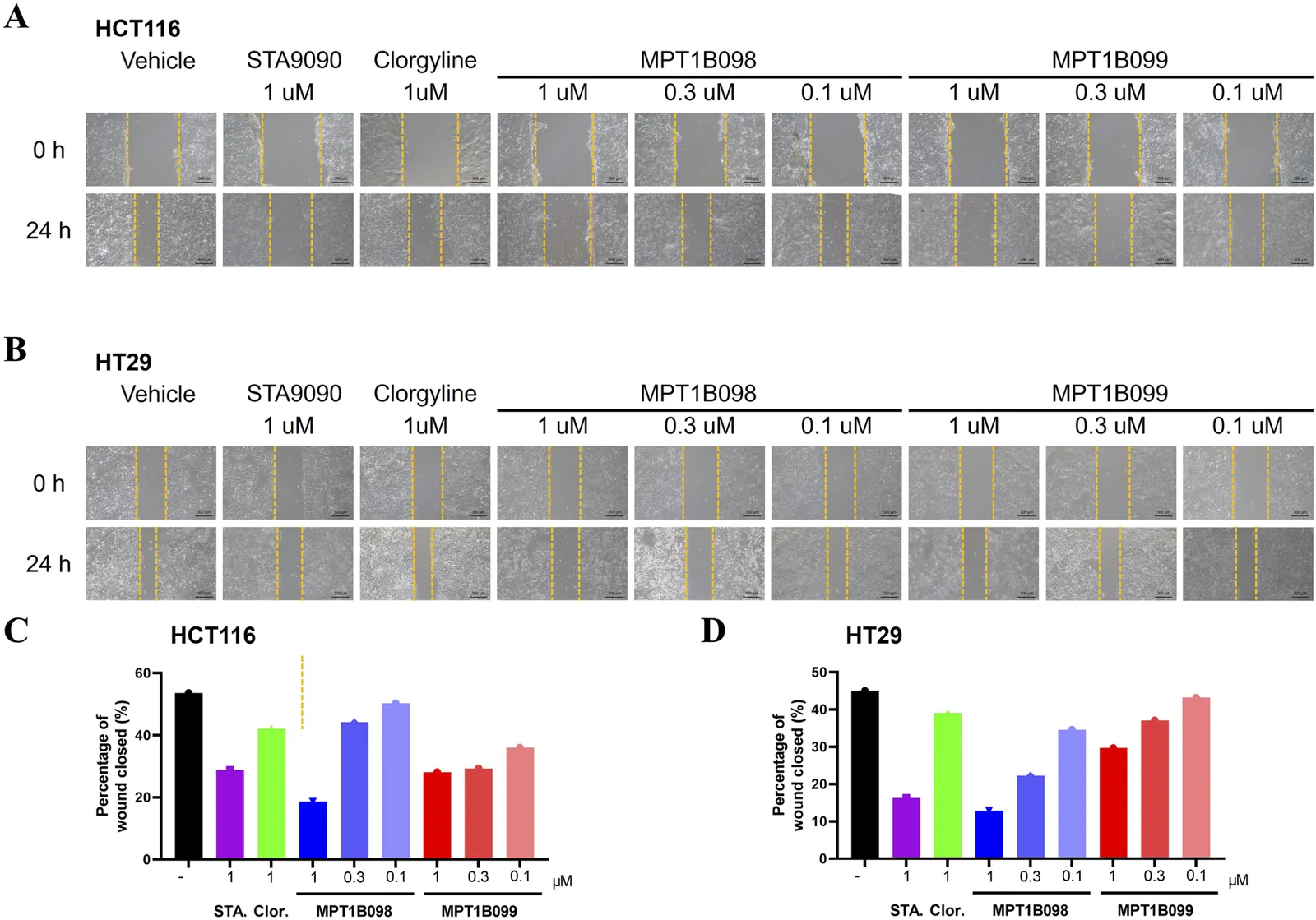
Effect of MPT1B098 and MPT1B099 on cell migration in colorectal cancers. **(A)** HCT116 and **(B)** HT29 were scratched with tips, treated with 10 ng/mL TGF-β and MPT1B098 or MPT1B099 at the indicated dose for 24h. **(C)** **(D)** The distance of the wound was measured and quantified in the bar graph.

MAO A has been reported to promote epithelial-to-mesenchymal transition (EMT) and play a pivotal role in facilitating cancer cell migration (Wu et al., 2014). Inhibition of MAO A ought to decrease cancer cell migration. To evaluate the anti-migration effect of MPT1B098 and MPT1B099, scratch assays were performed. Colorectal cancer cells, HCT116 and HT29, were seeded in 24-well plates, scratched with a pipette tip, and imaged following compound treatment. Cell migration was assessed by comparing images taken at time zero and 24 h post-scratching (Figure 5). In untreated cells, wound closure reached 53% in HCT116% and 45% in HT29. Treatment with compounds MPT1B098 and MPT1B099 both reduced wound closures. The percentage of wounds closed in MPT1B098-treated HCT116 cells decreased to 18.6% at 1 μM, 44.2% at 0.3 μM, and 50.3% at 0.1 μM; which were 12.9% at 1 μM, 22.3% at 0.3 μM, and 34.6% at 0.1 μM in HT29 cells. The percentage of wounds closed in MPT1B099-treated HCT116 cells decreased to 28.1% at 1 μM, 29.3% at 0.3 μM, and 36% at 0.1 μM; which were 29.7% at 1 μM, 37.1% at 0.3 μM, and 43.2% at 0.1 μM in HT29 cells. These results indicated that MPT1B098 and MPT1B099 effectively inhibited colorectal cancer cell migration in a dose-dependent manner.

### 3.5 Effects of MPT1B098 and MPT1B099 in downstream signaling

**FIGURE 6:**
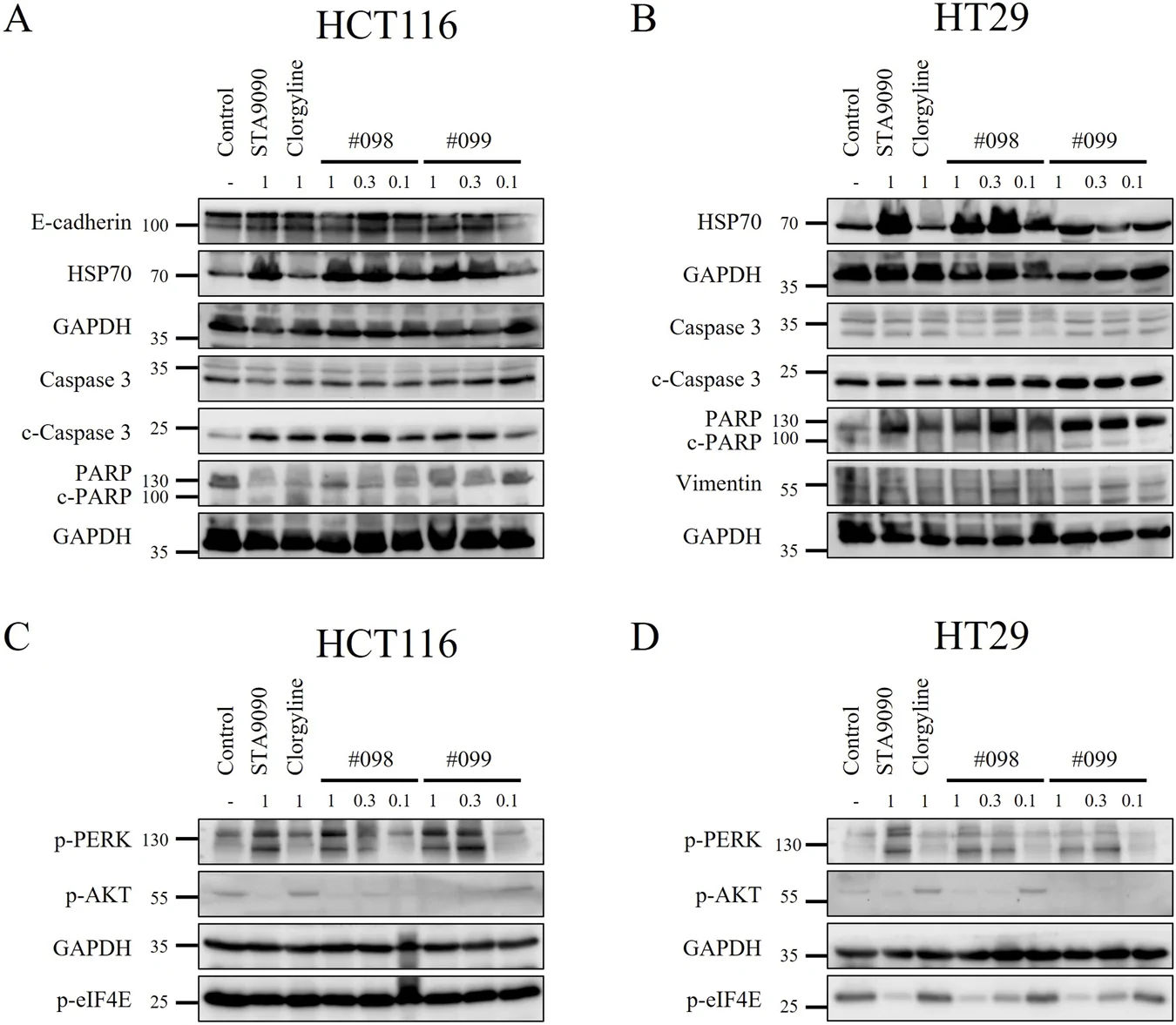
Effect of MPT1B098 and MPT1B099 on protein expression in colorectal cancer cells. **(A)** **(C)** HCT116 and **(B) (D)** HT29 cells were treated with MPT1B098, MPT1B099, and reference compounds at the indicated dose. The protein was collected and further analyzed by Western blot.

To investigate downstream signaling alterations in CRC cells following the treatment of MPT1B098 and MPT1B099, Western blot analysis was performed to assess key molecular markers associated with stress response, epithelial-mesenchymal transition (EMT), and apoptosis (Figure 6). After the treatment with MPT1B098 and MPT1B099, a marked upregulation of HSP70 was observed, consistent with a compensatory mechanism triggered by HSP90 inhibition. This elevation suggests cellular stress adaptation and highlights the dynamic interplay between chaperone proteins in maintaining proteostasis. E-cadherin, a critical epithelial marker, exhibited dose-dependent modulation with MPT1B099 treatment in HCT116 cells. At a higher concentration (1 μM), E-cadherin expression was increased compared with the control (no treatment), suggesting a potential reversal of EMT and restoration of epithelial characteristics. However, at higher doses, E-cadherin also underwent fractionation, which may reflect cytoskeletal remodeling or altered membrane localization under intensified stress conditions. In parallel, apoptotic markers were significantly elevated. Cleaved caspase-3 and cleaved PARP, both hallmarks of programmed cell death, were upregulated following treatment with MPT1B098 and MPT1B099, suggesting that dual inhibition effectively activates intrinsic apoptotic pathways.

HSP90 is a key chaperone protein; inhibition of HSP90 has been shown to enhance endothelial barrier function and simultaneously activate the unfolded protein response, thereby influencing the accumulation of unfolded proteins within the ER (Kubra et al., 2020). After the treatment with MPT1B098 or MPT1B099, a marked upregulation of p-PERK was observed, consistent with stress-associated activation of the PERK branch of the unfolded protein response, following HSP90 inhibition. The elevation of p-PERK further supports ER stress activation by HSP90 inhibition, suggesting that PERK-mediated signaling may contribute to the initiation of apoptosis (Taiyab et al., 2009). Phosphorylated Protein Kinase B (PKB, also known as AKT) is a key biomarker of active PI3K/AKT signaling, which regulates cell survival, growth, and metabolism (He et al., 2021). After treatment with MPT1B098 or MPT1B099, a significant reduction in p-AKT was observed, indicating inhibition of survival and metabolism signaling, and consistent with enhanced apoptotic activity in CRC cells. Eukaryotic translation initiation factor 4E (eIF4E) is the rate-limiting mRNA cap-binding protein in translation initiation. Phosphorylation of eIF4E activates the eIF4E complex, consequently driving selective translation of oncogenic mRNAs and promoting cell survival, tumor growth, and metastasis (Martineau et al., 2013; Chen et al., 2023). Interestingly, only HT29 exhibited a dose-dependent reduction in p-eIF4E, whereas HCT116 cells maintained stable p-eIF4E levels despite treatment. This differential regulation suggested that HT29 cells are more vulnerable to translational repression following HSP90 inhibition, while HCT116 cells rely on alternative signaling mechanisms to sustain downstream translation. Collectively, these findings demonstrate that MPT1B098 and MPT1B099 disrupt proteostasis, trigger stress adaptation, modulate EMT markers, and induce apoptotic signaling, while also revealing oncogenic dependencies that CRC cells respond to treatment.

### 3.6 MPT1B098 and MPT1B099 decrease the PD-L1 expression

The PD-1/PD-L1 signaling axis regulates both the initiation and maintenance of immune tolerance within the tumor microenvironment. PD-L1 is overexpressed in various cancer cells to escape immune cell recognition (Han et al., 2020). Multiple proinflammatory molecules in the tumor microenvironment could increase PD-L1 expression, including interferon-γ (IFN-γ), IFN-α, IFN-β, interleukin-6 (IL-6), IL-10, tumor necrosis factor-α (TNF-α), and microRNA (miRNA) (Yi et al., 2021). Among these, IFN-γ is widely recognized as the principal inducer of PD-L1 expression, activating the JAK-STAT signaling pathway to drive its expression (Yi et al., 2021; Zerdes et al., 2018). HSP90 inhibitors have been shown to reduce PD-L1 expression, thereby enhancing T cell activation (Zavareh et al., 2021). Similarly, deletion of MAO A alleviates immune suppression and promotes cytotoxic T cell activity, particularly in prostate cancer (Lapierre et al., 2022). Previously, dual MAO A/HSP90 inhibitors MPT1B098 and MPT1B099 were shown to suppress IFN-γ-induced PD-L1 expression in a concentration-dependent manner in glioblastoma cells GL26. These findings suggest that MPT1B098 and MPT1B099 may also reduce PD-L1 expression in CRC, potentially overcoming immune resistance and enhancing the efficacy of checkpoint blockade. Herein, their effects in HCT116 (MSI) cell lines were also evaluated, the results demonstrated their potential in decreasing PD-L1 expression in HCT116, and were presented in the supplementary information in the previous publication (Tseng et al., 2023). However, their effects in HT29 (MSS) have not yet been revealed.

**FIGURE 7:**
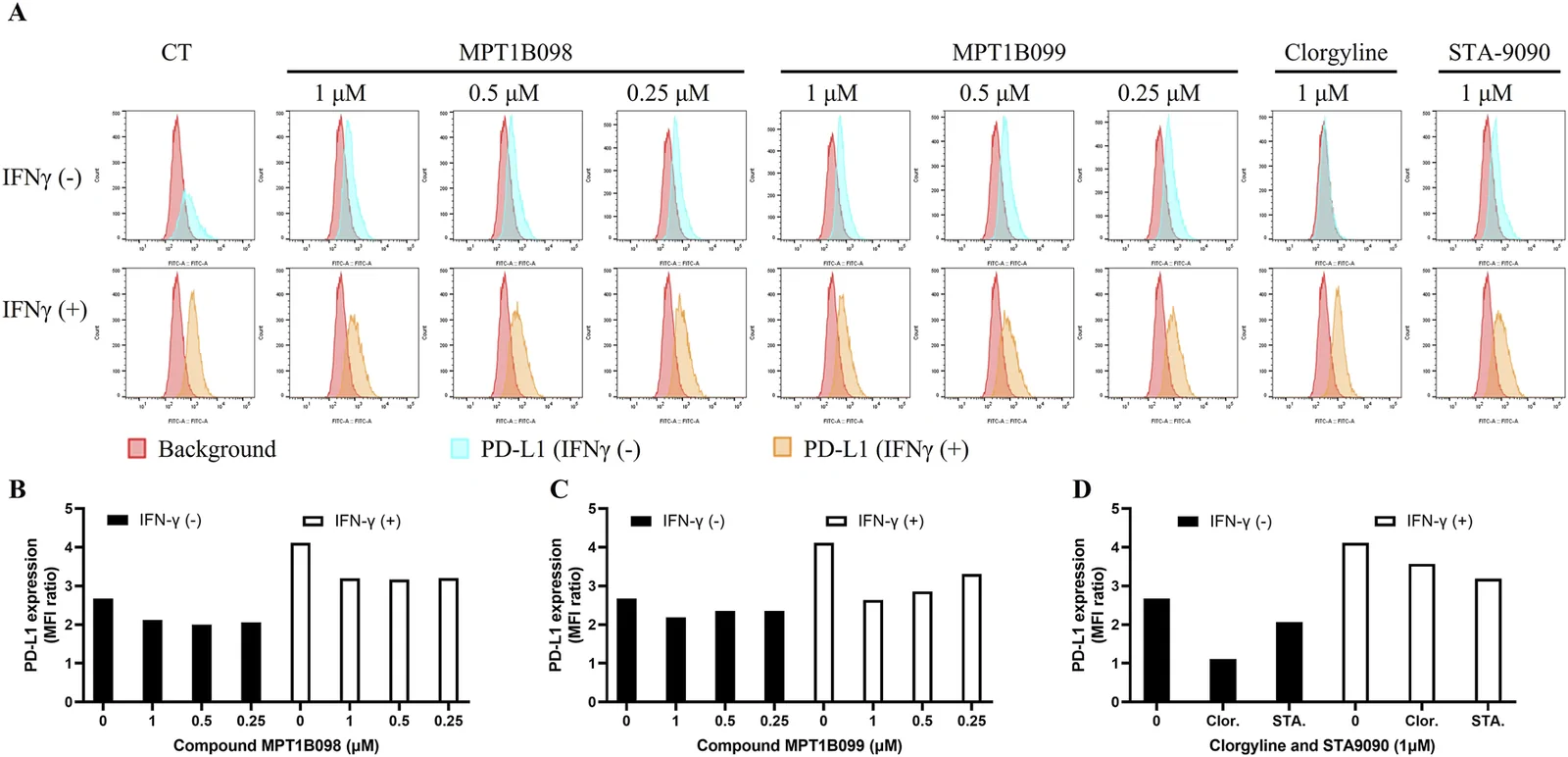
Effects of MPT1B098 and MPT1B099 on decreasing PD-L1 expression in colorectal cancer cells. HT29 were treated with MPT1B098, MPT1B099, clorgyline, or STA-9090 at various concentrations with or without 10 ng/mL IFN-γ for 24 h **(A)** PD-L1 expression was determined using flow cytometry analysis and represented as fluorescence intensity. **(B–D)** The mean fluorescence intensity (MFI) of each sample after normalization to unstained background were represented as MFI ratio using bar figures, **(B)** MPT1B098, **(C)** MPT1B099, and **(D)** clorgyline and STA-9090.

To evaluate the effect of MPT1B098 and MPT1B099 on PD-L1 expression in the HT29 cell line, HT29 cells were treated with MPT1B098, MPT1B099, clorgyline, or STA-9090 at the indicated concentrations for 24 h with or without 10 ng/mL of INF-γ. After 24 h, PD-L1 expression was determined (Figure 7). Results showed that both MPT1B098 and MPT1B099 decrease PD-L1 expression with similar potency. The parent compounds, clorgyline and STA-9090, were less effective at high concentration. These results indicated that MPT1B098 and MPT1B099 demonstrated potential as immune checkpoint inhibitors.

### 3.7 Dose tolerance of MPT1B098 and MPT1B099 in mice

**FIGURE 8:**
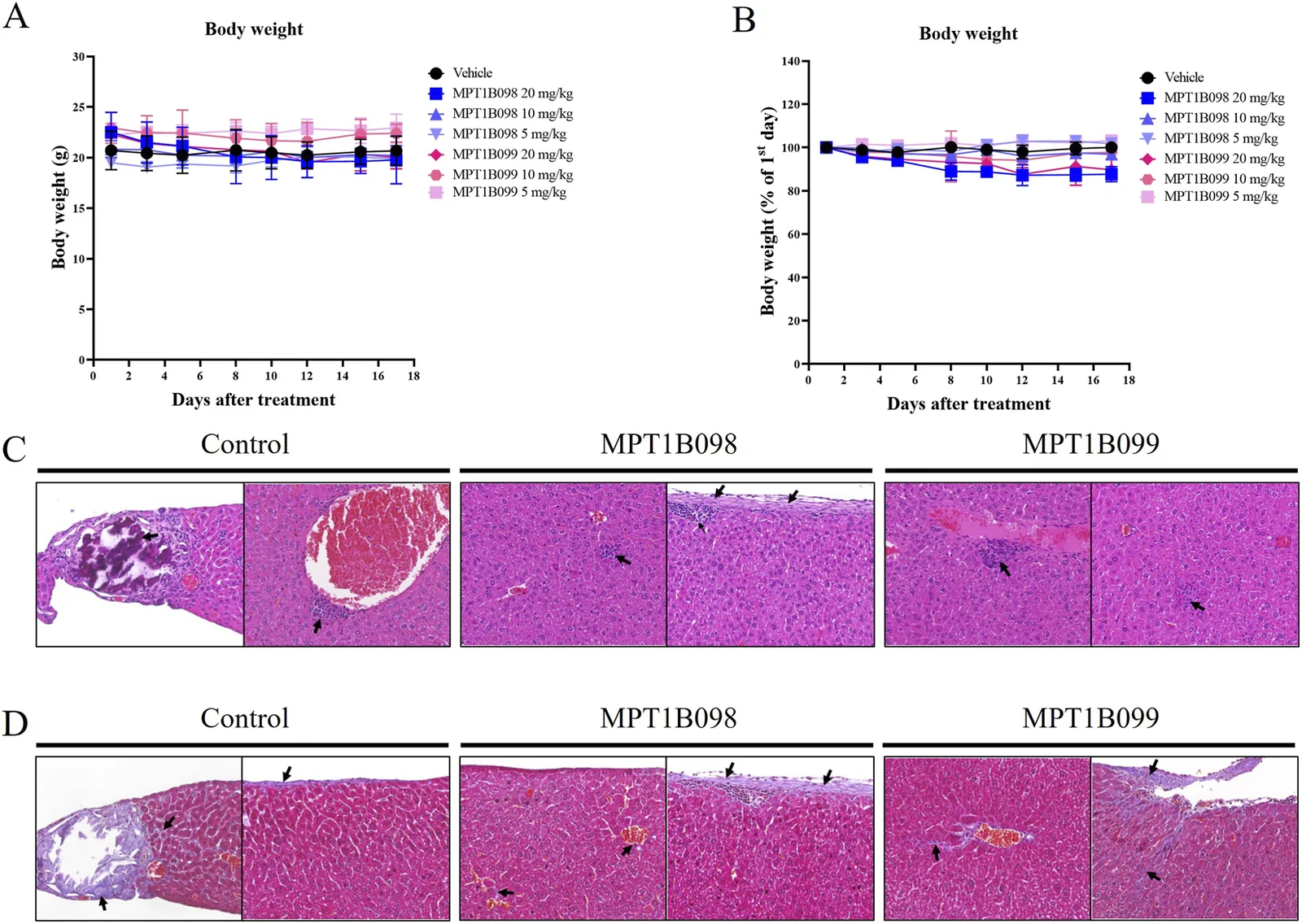
Body weight and pathological examination of the liver tissues from MPT1B098 and MPT1B099 treated mice. Mice were treated with vehicle, MPT1B098 or MPT1B099 at 5, 10, 20 mg/kg three times per week. Their body weight was recorded and presented as **(A)** Body weight (g) and **(B)** Body weight (% of first day). Their liver tissue slides were stained with **(C)** hematoxylin and eosin (H&E) and **(D)** Masson’s trichrome (TRI) stain.

To select an optimized dosage for cancer treatment in murine models, three doses were tried in BALB/c mice. The results indicated that mice showed good tolerance to 5 and 10 mg/kg of MPT1B098 or MPT1B099 when treated three times per week via intraperitoneal injection. The body weight of mice decreased in the 20 mg/kg MPT1B098 or MPT1B099 treated group (Figure 8). The histopathological examination on the liver of control, 20 mg/kg MPT1B098, or MPT1B099 treated mice was also analyzed (Figure 8 and Table 1). The result indicated that the liver showed minimal inflammatory cell infiltration; chronic inflammation with mineralization was not observed; capsular chronic inflammation was minimal to slight; and hepatic fibrosis (collagen deposition) in the hepatic capsule and/or hepatic parenchyma was minimal. Although several microscopic lesions were observed in the live tissue, they are considered spontaneous. Herein, we chose the dose of 10 mg/kg for the following murine model study.

**TABLE 1:** Histopathology finding table.

Group	Vehicle			MPT1B098			MPT1B099		
Animal ID	V-1	V-2	V-3	M8-1	M8-2	M8-3	M9-1	M9-2	M9-3
Inflammatory cell infiltration	1	1	1	1	1	1	1	1	1
Chronic inflammation with mineralization	1	0	0	0	0	0	0	0	0
Capsular chronic inflammation	0	2	1	0	0	2	1	1	1
Hepatocellular necrosis	0	0	0	0	0	0	0	0	0
Fibrosis (Masson’s trichrome stain)	1	1	1	0	0	1	1	1	1

### 3.8 MPT1B098 and MPT1B099 suppress tumor growth in MSI and MSS colorectal cancer murine model in combination with anti-PD-1 therapy

**FIGURE 9:**
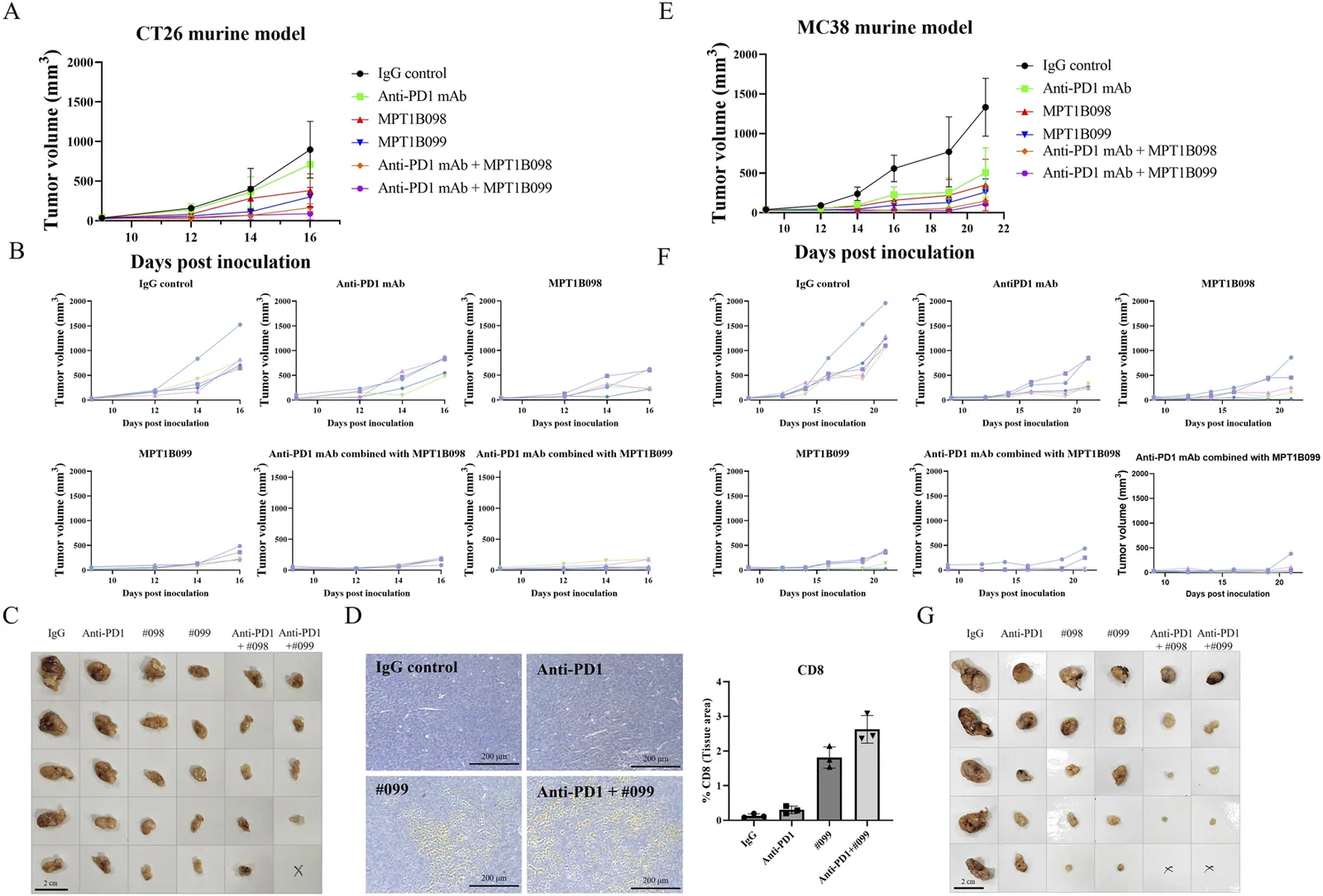
The anti-tumor growth effect of MPT1B098 and MPT1B099 in the MC38 and CT26 murine colorectal cancer model. **(A–D)** BALB/c mice were injected with CT26 cancer cells in their back. **(E–G)** C57BL/6 mice were injected with MC38 cancer cells. After tumor size reached 50 mm^3^, mice were treated with 100 ug/kg IgG control as the vehicle, 100 ug/kg anti-PD1 antibodies, 10 mg/kg MPT1B098, 10 mg/kg MPT1B099, or a combination of anti-PD1 antibodies and MPT1B098 or MPT1B099. Tumor size was measured with a clipper three times per week. The average tumor size of each treatment group of the CT26 model **(A)** and the MC38 model **(E)**. The tumor size of individual mice of each treatment group of the CT26 model **(B)** and the MC38 model **(F)**. The tumor volume of each treatment group of the CT26 model **(C)** and the MC38 model **(G)**. **(D)** The CD8 signal in tumor tissues after treatment of MPT1B099 and its combination in the CT26 murine colorectal cancer model. The tumor tissues were harvested, fixed in formalin, paraffin-embedded, sectioned, and coated on slides. The tissue sections were deparaffinized, stained with anti-CD8 antibodies, and counterstained with hematoxylin.

To evaluate tumor growth inhibition of MPT1B098 and MPT1B099 in MSI and MSS CRC, murine models of MC38, representing MSI, and CT26, representing MSS, were employed (Figure 9). MC38 cells were implanted subcutaneously into C57BL/6 mice, while CT26 cells were implanted into BALB/c mice. After the tumor size reached 50 mm^3^, mice were treated with IgG control, anti-PD-1 antibodies, MPT1B098, MPT1B099, or a combination of MPT1B098 or MPT1B099 with anti-PD-1 antibodies. As expected, anti-PD-1 antibody monotherapy suppressed tumor growth only in the MC38 (MSI) model, but not in the CT26 (MSS) model (Figures 9A-C,E-G). In CT26 (MSS) tumors, the limited efficacy of anti-PD-1 monotherapy reflects their low immunogenicity and poor baseline T-cell infiltration, which restricts checkpoint blockade activity. In contrast, both MPT1B098 and MPT1B099 significantly suppressed tumor growth in both models, and the combination therapy further enhanced suppression. However, the synergistic effect was barely observed, likely because MPT1B098 or MPT1B099 alone already had strong efficacy.

MPT1B098 and MPT1B099 were found to decrease PD-L1 expression in both HT29 (Figure 7) and HCT116 cells (Tseng et al., 2023). However, they showed only a modest synergistic effect when combined with anti-PD1 antibodies in both the CT26 and MC38 models. This effect may be attributed to their modulation in the immune microenvironment. Immunohistochemistry of CT26 tumors revealed that MPT1B099 elevated CD8^+^ T-cell infiltration and (Figure 9D) its combination with anti-PD1 antibodies further increased it. Consistent with the poor immunogenicity of MSS tumors, anti-PD-1 monotherapy alone was ineffective. However, MPT1B099 not only suppressed tumor growth but also increased CD8^+^ T cell infiltration, partially sensitizing MSS tumors to PD-1 blockade. This may be due to decreased PD-L1 expression induced by MPT1B099, which, in turn, decreased the activity of T regulatory cells. These results indicated their potential anti-tumor growth effect in MSI- and MSS-type CRC. Despite only a mild synergistic effect, these results demonstrate that the compounds exert potent antitumor and immune-modulatory effects, overcoming key resistance features of MSS CRC.

## 4 Discussion

In this study, the effects of the dual MAO A/HSP90 inhibitors MPT1B098 and MPTB099 were evaluated in colorectal cancer. Both compounds demonstrated significant potency against CRC cells by reducing cell viability and migration, inducing apoptosis, blocking cell cycle progression at the G2/M phase, modulating protein expression, and suppressing tumor growth *in vivo*. These findings positioned dual MAO A/HSP90 inhibitors as promising therapeutic agents.

Despite considerable progress in developing small-molecule immune checkpoint inhibitors, only a few have received clinical approval. Nevertheless, several candidates are currently in clinical trials, and combination strategies have already demonstrated promising outcomes (Fuchs et al., 2024; Ge et al., 2024). Quemliclustat is a small-molecule CD73 inhibitor granted orphan drug designation for pancreatic cancer in 2025 (Piovesan et al., 2022; Cortese, 2025). Another CD73 inhibitor, LY3475070, has been combined with pembrolizumab to treat advanced cancer and is under a phase I clinical trial (Bi et al., 2025). VISTA inhibitor, SNS-101, is currently under a phase I clinical trial for refractory cancer (Thisted et al., 2024). Collectively, these revealed the emerging potential of small molecules as immune checkpoint-targeted therapies. Building on this concept, our previous work was inspired by the study by Zavareh et al., which demonstrated that HSP90 inhibition downregulates PD-L1 expression (Zavareh et al., 2021). Accordingly, MPT1B098 and MPTB099 have been developed and found to effectively reduce PD-L1 expression in glioblastoma and colorectal cancer cells (Tseng et al., 2023).

In this study, two human and two murine CRC cell lines were used to compare the compound’s efficacy across different gene and microsatellite stability states in CRC. HCT116, human CRC cells, have a wild-type p53 tumor suppressor gene and a mutated K-Ras oncogene. Further, HCT116 has a lower level of the mismatch repair (dMMR) gene and exhibits microsatellite instability (MSI) (Gayet et al., 2001). MC38, murine CRC cells, have characteristics similar to those of HCT116. MC38 also possesses wild-type p53, has reduced levels of the MMR genes, and exhibits MSI (Thomas et al., 2023). But, MC38 expresses wild-type K-Ras (Liu et al., 2023). In contrast, HT29 has a mutated p53 gene and a wild-type K-Ras oncogene. HT29 has a higher level of the MMR gene (pMMR) and shows microsatellite stability (MSS) (Gayet et al., 2001). CT26, murine CRC cells, only have partially similar characteristics to HT29. CT26 shows a higher level of MMR and exhibits MSS. While CT26 exhibits a wild-type p53 gene and a mutated K-Ras (Wu et al., 2024). In the MTT assays, STA-9090 (Ganetespib), a known HSP90 inhibitor, was used as a reference compound. The result revealed that MPT1B098 and MPT1B099 exhibited inferior efficacy in HCT116 and HT29 compared to STA-9090. This might be due to the structure modification, which removes the five-membered heterocycle and replaces it with clorgyline (Figure 1), potentially altering the compound’s binding affinity to HSP90 and reducing its cytotoxic potency *in vitro*. Interestingly, MPT1B098 and MPT1B099 exhibited superior cytotoxicity in the murine colorectal cancer cell lines MC38 and CT26 compared to STA-9090 and clorgyline. The apoptosis assay and cell cycle distribution further support the potential cytotoxic effect. In the scratch assay, the percentage of wound closure supported the effect of MPT1B098 and MPT1B099 in the inhibition of cell migration, which is similar to the mother structure clorgyline. The Western blot further confirmed their effects on EMI and apoptosis markers.

Although most downstream markers responded similarly in both CRC cell lines, western analysis revealed a striking divergence in p-eIF4E regulation, revealing cell line-specific differences in translational control (Figure 6). The different effects of MPT1B098 and MPT1B099 on p-eIF4E between HCT116 and HT29 cells likely reflect their distinct oncogenic drivers. HT29 cells with BRAF mutations were highly dependent on MAPK/ERK signaling and exhibited a dose-dependent reduction in p-eIF4E, indicating that translational repression may represent a critical vulnerability after HSP90 inhibition. In contrast, HCT116 cells with KRAS mutations and strong PI3K/AKT activity maintained p-eIF4E but reduced p-AKT, suggesting that HSP90 inhibition was buffered and p-eIF4E levels were preserved (Troiani et al., 2014; Valcikova et al., 2024). These results revealed the cell line-specific signaling responses. Both HT29 and HCT116 showed decreased p-AKT after treatment, but only HT29 demonstrated greater sensitivity through downregulation of p-EIF4E, highlighting distinct oncogenic dependencies in CRC cells.

To assess the safety of MPT1B098 and MPT1B099, a dose-tolerance study was conducted in mice. The primary data already showed a decline in the 20 mg/kg group (Figure 8). Although the following study used 10 mg/kg to evaluate efficacy, it is suggested that the maximum tolerated dose and acute toxicity tests be confirmed. In our previous study, MPT1B098 and MPT1B099 reduced PD-L1 expression in HCT116 cells and here, we also demonstrated that MPT1B098 and MPT1B099 decrease PD-L1 expression in HT29 cells (Figure 7). These may explain the increased CD8^+^ T cell infiltration observed in this study (Figure 9D) (Tseng et al., 2023). PD-L1 downregulation can relieve immunosuppression by diminishing Treg activity and restoring cytotoxic T cell function, thereby enhancing antitumor immunity. Notably, MPT1B099, when combined with anti-PD1 antibodies, further elevated CD8 signals, supporting a synergistic effect. To strengthen this mechanistic link, future studies should evaluate functional markers of CD8^+^ T cell activity, such as granzyme B, which have been widely used in preclinical studies of checkpoint inhibitors, such as nivolumab, to confirm enhanced cytotoxicity (Hurkmans et al., 2020). Additional approaches, including flow cytometry to characterize CD8^+^ T cell subsets and multiplex immunofluorescence to assess spatial distribution, will be crucial in determining whether PD-L1 reduction by MPT1B098 and MPT1B099 translates into robust CD8^+^ T cell-mediated tumor killing. Further, while the murine models provide valuable insights, they may not fully recapitulate the complexity of human CRC. Further pharmacokinetic and pharmacodynamic profiling will be essential to assess the suitability of MPT1B098 and MPTB099 for clinical development. Their potential to synergize with existing immunotherapies, such as anti-PD1 or anti-CTLA-4 antibodies, should be explored in more immunocompetent orthotopic CRC models.

**FIGURE 10:**
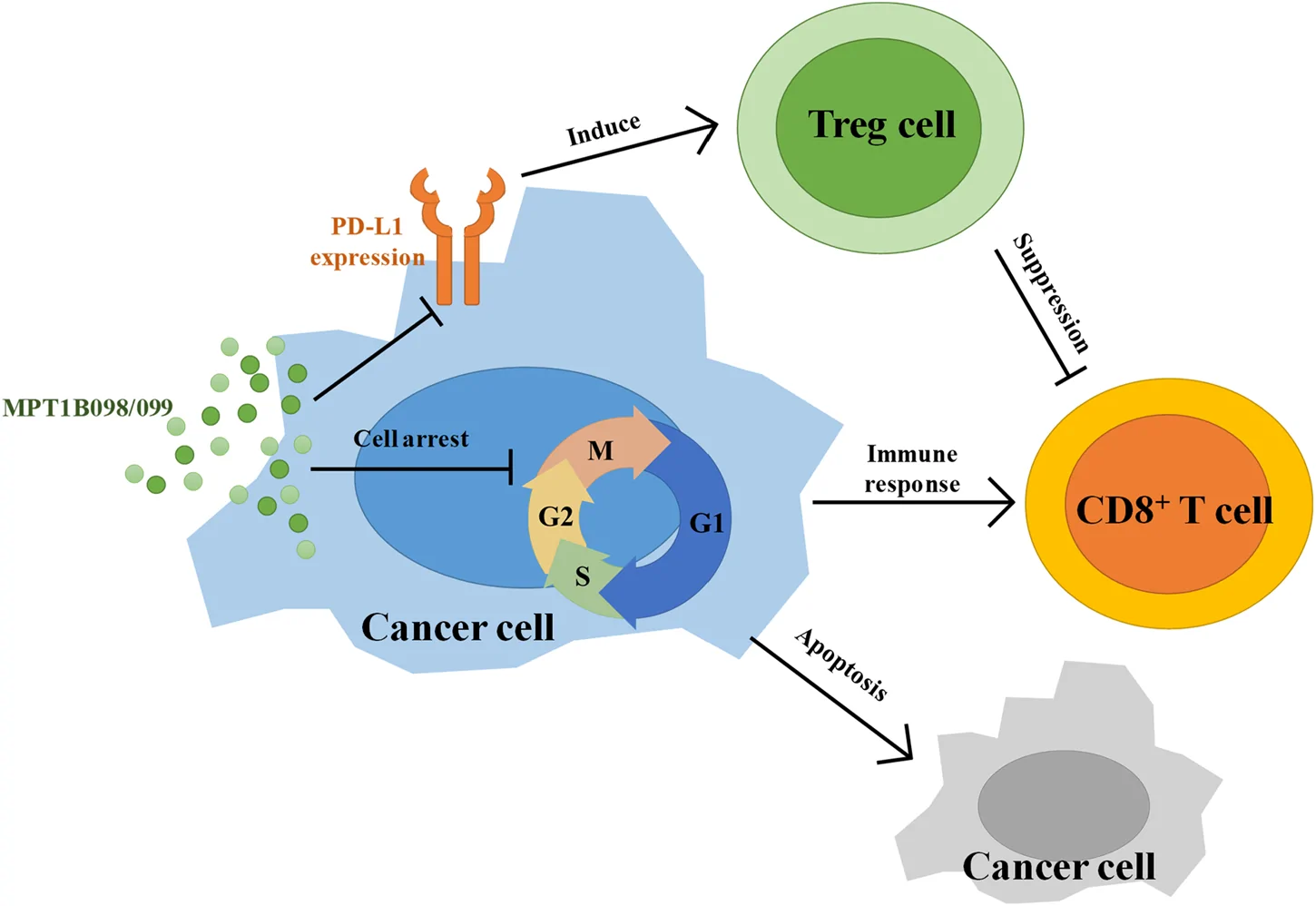
Mechanistic overview of MPT1B098 and MPT1B099 in colorectal cancer. MPT1B098 and MPT1B099 downregulate PD-L1 expression, which might reduce immune evasion and suppress regulatory T cells, and thus enhance CD8^+^ T cell infiltration into the tumor microenvironment. They also induce cell cycle arrest at the G2/M phase and trigger apoptosis. These collectively contributed to the antitumor and immune-modulatory effects.

To our knowledge, MPT1B099 represents the first MAO A and HSP90 inhibitor shown to enhance CD8^+^ T cells infiltration in CRC models, presenting a novel strategy to overcome immune resistance in MSS tumors. Given the role of MAO A and HSP90 in multiple solid malignancies, these dual-targeting compounds may have therapeutic relevance beyond CRC, particularly in tumors characterized by immune evasion and resistance to checkpoint blockade. Collectively, our findings support the therapeutic potential of MPT1B098 and MPT1B099 as multifunctional agents that can disrupt tumor survival pathways while simultaneously promoting antitumor immunity (Figure 10). These mechanistic insights provide a compelling rationale for dual inhibition strategies to overcome resistance and enhance the efficacy of cancer immunotherapy.

## 5 Conclusion

This study evaluated the effects of dual MAO A/HSP90 inhibitors, MPT1B098 and MPT1B099, which demonstrated multifaceted anti-tumor activity in both MSI and MSS colorectal cancer models. *In vitro*, both compounds reduced cell viability in murine and human colorectal cancer cell lines, with sub-micromolar IC_50_ values. They also inhibited cancer cell migration, induced apoptosis, and modulated cell cycle progression. Western blot analysis confirmed their effects on downstream signaling pathways of the unfolded protein response, as well as on translational, apoptotic, and EMT-related markers. *In vivo*, MPT1B098 and MPT1B099 were well tolerated in BALB/c mice at doses ranging from 5 to 20 mg/kg, administered intraperitoneally three times per week and histological examination of liver tissues supported the safety of the selected 10 mg/kg dose for further studies. In murine colorectal cancer models, both compounds significantly suppressed tumor growth in MSI (MC38) and MSS (CT26) tumors. While anti-PD-1 monotherapy was effective only in the MSI model, MPT1B098 and MPT1B099 demonstrated efficacy in both. Combination therapy with anti-PD-1 antibodies showed a slightly synergistic effect in both models, suggesting that dual MAO A/HSP90 inhibition may sensitize tumors to immune checkpoint blockade. Immunohistochemistry analysis further revealed increased infiltration of CD8^+^ cytotoxic T cells in the tumor microenvironment following treatment with MPT1B098, particularly when combined with anti-PD-1 antibodies. This immune activation may be attributed to reduced PD-L1 expression and suppression of regulatory T cell activity, highlighting the immune-modulatory potential of these compounds.

## Data Availability

The original contributions presented in the study are included in the article/supplementary material, further inquiries can be directed to the corresponding authors.
